# Editorial for the Special Issue Applications of Nanomaterials in Plasmonic Sensors

**DOI:** 10.3390/nano12101634

**Published:** 2022-05-11

**Authors:** Oleg Vitrik

**Affiliations:** 1 Institute of Automation and Control Processes (IACP) FEB RAS 1, 690041 Vladivostok, Russia; oleg_vitrik@mail.ru; 2 Photonics and Digital Laser Technologies Department, Far Eastern Federal University (FEFU), 690922 Vladivostok, Russia

Further progress in the modern sensor industry is associated with the widespread application of new solutions and principles from the field of nanooptics and nanophotonics. In this regard, the idea of using surface plasmon resonance (SPR) for physical measurements has proven to be most fruitful. SPR is the resonant excitation of surface plasmons, which are coupled oscillations of the electromagnetic field and conduction electrons at the interface between a dielectric and a conductor. They exist in two main forms, propagating surface plasmon polaritons (SPPs) and localized surface plasmons (LSPs). An SPP can be interpreted as a guided mode of a special waveguide, which is a sufficiently extended (in one or two dimensions) interface between a noble metal and dielectric. LSPs are the modes of subwavelength resonators, which are plasmonic nanoparticles and nanostructures of various shapes [1,2,3,4].

The spectral response of the SPR is easily modulated by the slightest changes in the properties of the dielectric medium adjacent to the plasmonic waveguide or resonator. This turns out to be extremely important from the point of view of physical measurements and allows for the creation of SPR sensors for environmental parameters, primarily for the refractive index. The refractive index can change, for example, due to the binding or dissociation of the target analyte molecules, or other chemical or physical processes, which is thus detected by the SPR sensor. In other words, in the classical case such devices are no more than refractometers, but are capable of ultra-sensitive, label-free measurements [5,6,7,8,9,10]. Otto and Kretschmann configurations became the classical optical schemes for such devices. Proposed back in 1968, they are still used today, albeit with many changes, variations, and improvements to create sensor platforms for chemical and biochemical analysis, environmental monitoring, food safety, medical diagnostics, and other similar applications [11,12].

SPR may not be a direct "detector" for the presence of biomolecules or molecules of other analytes, but nevertheless, plays an extremely important role in their detection. The classic technique in this respect is the use of nanorough plasmonic surfaces. The high strength of electric fields localized near the features of the nanorelief of such surfaces, due to the excitation of LSPs in them, along with the charge transfer mechanism, makes it possible to achieve a huge enhancement of the Raman scattering. This approach, known as surface-enhanced Raman spectroscopy (SERS), was first proposed by Martin Fleischmann, Patrick J. Hendra and A. James McQuillan in 1973 [13]. It since became the basis for methods of detecting analytes in solutions of extremely low concentrations, up to single molecule SERS detection [14,15,16,17].

Classical schemes of Otto and Kretschmann refractometers, as well as SERS systems, became the basis of some commercially available instruments. The ongoing work to improve their sensitivity, selectivity, and other measurement characteristics, in addition to improving ergonomics and reducing size, weight, and cost, can be attributed to applied engineering research.

As for the fundamental aspects of the development of plasmonic sensors, the current research activities include searching and studying new plasmonic materials for such devices (new bulk materials, such as plasmonic semiconductors and hybrid structures [18,19,20,21], and low-dimensional materials such as 2D graphene layers, 1D nanotubes and 0D quantum dots) [22,23,24,25,26,27,28]. Also, studies are being carried out on the SPR excitation in resonators of complex geometry and on the interaction between various plasmonic nanostructures [28]. This is especially relevant to regular nanostructured systems called metamaterials, whose collective response differs significantly from the response of its individual structural units [29,30,31]. Increasingly, considerable attention is paid to peripheral components of plasmonic sensors, such as analyte concentrators, which, although not possessing the properties of a sensitive element, are nevertheless capable of increasing the sensitivity of sensors by several orders of magnitude [32,33,34,35]. In addition, of course, all this requires the study of technological issues of nanofabrication of the corresponding elements and structures. Taking advantage of these new materials and approaches, one can design plasmonic sensors with unique metrological properties.

Despite the fact that this Special Issue contains only six articles, it nevertheless touches in one way or another on all the above-mentioned topical trends in the development of the principles of plasmonic sensors.

The issue covers new multi-layer substrates for SERS with an increased enhancement factor relative to conventional Raman spectroscopy; a new 3D metamaterial capable of supporting an ultra-narrowband hybrid plasmon mode, which potentially provides ultrahigh sensing performance characteristics for biomedical sensors; a new 2D plasmonic material, borophene, for sensing applications; a new approach to the analyte enrichment, based on the effect of a non-uniform electrostatic field on the evaporating droplet; an alternative simple analytical approach to calculate the SPPs amplitudes, which can be useful for calculating the parameters of plasmonic elements; and a technology for the synthesis of ZnO nanorod arrays for UV detectors and other applications.
